# Phenethyl Isothiocyanate Induces Apoptosis Through ROS Generation and Caspase-3 Activation in Cervical Cancer Cells

**DOI:** 10.3389/fphar.2021.673103

**Published:** 2021-07-29

**Authors:** Shoaib Shoaib, Saba Tufail, Mohammad Asif Sherwani, Nabiha Yusuf, Najmul Islam

**Affiliations:** ^1^ Department of Biochemistry, J.N.M.C, Aligarh Muslim University, Aligarh, India; ^2^ Department of Dermatology, University of Alabama at Birmingham, Birmingham, AL, United States

**Keywords:** cervical cancer, HPV16+, ROS, apoptosis, DNA fragmentation

## Abstract

The latest research shows that current chemotherapeutics are ineffective because of the development of resistance in cervical cancer cells, and hence, their scope of use is limited. The main concern of researchers at the moment is the discovery of safe and effective antiproliferative plant chemicals that can aid in the battle against cervical cancer. Previous studies have shown the possible anticancer potential of phenethyl isothiocyanate obtained from cruciferous plants for many cancers, which targets various signaling pathways to exercise chemopreventive and therapeutic effects. This provides the basis for studying phenethyl isothiocyanate's therapeutic potential against cervical cancer. In the present study, cervical cancer cells were treated with various doses of phenethyl isothiocyanate, alone and in combination with cisplatin. Phenethyl isothiocyanate alone was sufficient to cause nucleus condensation and fragmentation and induce apoptosis in cervical cancer cells, but evident synergistic effects were observed in combination with cisplatin. In addition, phenethyl isothiocyanate treatment increased the production of intracellular ROS in a dose-dependent manner in cervical cancer cells. Furthermore, investigation of phenethyl isothiocyanate induced mitochondrial reactive oxygen species production, and activation of caspases showed that phenethyl isothiocyanate significantly activated caspase-3.

## Introduction

Being one of the critical and major public health problems, cervical cancer stands fourth in women and seventh among all the cancers in the world (Ferlay et al., 2015). Epidemiological studies have indicated its high incidence and mortality, accounting for 0.57 million cases and 0.311 million deaths in 2018 (Cohen et al., 2019). In most cervical cancer malignancies, human papilloma virus (HPV) infection was found to be related to HPV16 and HPV18 at higher risk (Moody and Laimins, 2010). Although medical facilities have progressed, cervical cancer is still rising in terms of incidence and deaths worldwide due to the lack of cost-effective diagnosis and therapies (Arbyn et al., 2011).

The most common cervical cancer treatment strategies are hysterectomy, radiotherapy, chemotherapy, and chemoradiotherapy. The surgery is successful if cervical cancer is diagnosed early, but largely, metastasized cervical cancer is diagnosed at the late stage (Sak, K., 2014). Radiation therapy of patients with cervical cancer or any other cancer has been reported for adverse side effects on the gastrointestinal, gynecological, and urological systems (Klee et al., 2000). The concurrent use of chemotherapy combined with radiotherapy has also been reported to give rise to hematologic and gastrointestinal side effects in cervical cancer (Eifel, 2006). The standard first-line medicaments (chemotherapeutics) for the treatment of cervical cancer are cisplatin and 5-fluorouracil–based chemotherapy; however, it is accompanied by drug resistance. Reckoning with these conditions, the development of a safe treatment plan, which is more precise and effective, with the least side effects is urgently required.

In recent decades, cervical cancer has been targeted by a variety of herbal compounds for their anticancer potential, creating opportunities to discover new anticancer medicines to address the problems of drug resistance and adverse side effects caused by traditional therapies. Multiple herbal compounds have been shown to have important effects on various signaling pathways of the cervical cancer cell (Chauhan et al., 2009; Moga et al., 2016). Phenethyl isothiocyanate (PEITC), most commonly seen in watercress and cruciferous vegetables, exhibits various beneficial behaviors. In cancer cells, PEITC has been shown to have antioxidant, anti-inflammatory, and antiproliferative activity and induces apoptosis (Xu et al., 2005; Satyan et al., 2006; Hong et al., 2015; Sarkar et al., 2016; Ramirez et al., 2018). In the current study, we tried to investigate the effects of PEITC on the HPV16+ and HPV18+ cervical cancer cell lines (CaSki and HeLa).

## Materials and Methods

### Reagents

All the reagents used in this study were of molecular grade. PEITC, cisplatin, propidium iodide (PI), 4,6-diamidino-2-phenylindole (DAPI), 2,7-dichlorofluorescein diacetate(DCF-DA), dihydrorhodamine 123, N-acetylcysteine (NAS), 3-(4,5-dimethylthiazol-2-yl)-2,5-diphenyl tetrazolium bromide (MTT), dimethyl sulfoxide, and caspase-3, -8, and -9 inhibitors were procured from Sigma Aldrich Chemical Co. (United States). Caspase activity kits were purchased from BioVision, United States, and an FITC Annexin V Apoptosis kit was obtained from BD Biosciences (United States). Fetal bovine serum (FBS), Dulbecco’s modified Eagle’s medium (DMEM), and other chemicals were purchased from Thermo Fisher Scientific.

### Cell Culture

Human cervical cancer cell lines (CaSki and HeLa) and a normal human keratinocyte cell line (HaCaT) were purchased from the National Center for Cell Sciences (NCCS), Pune, India and ATCC, respectively. The cells were grown in DMEM supplemented with 10% FBS and 1% antibiotic–antimycotic solution containing penicillin and streptomycin. The cells were maintained in a humidified atmosphere containing 5% CO_2_ at 37°C in a CO_2_ incubator.

### Determination of Cell Viability Using MTT Assay

To determine the effect of phenethyl isothiocyanate on normal human keratinocyte, HaCaT cells and cervical cancer cells (CaSki and HeLa) were analyzed using MTT assay. Briefly, the cells were seeded into a 96-well plate at a density of 5 × 10^3^ cells per well in triplicate and incubated at 37°C for 24 h. The cells were treated with different concentrations of PEITC (0, 2, 5, 10, 15, 20, and 25 µM) for 24 and 48 h. Control group cells were treated with 0.01% DMSO in media. Subsequently, cell survival was determined by adding 10 µL of 5 mg/ml MTT reagent in each well, followed by incubation for 4 h at 37°C, and then 100 µL of dimethyl sulfoxide was added to dissolve formazan by gentle shaking. The absorbance of each well was measured at 570 nm, and cell survival was calculated as percentage over the control. Additionally, cisplatin was also used to determine and compare cytotoxicity on the cell lines, either alone or in combination with the tested compound. The purpose of including HaCaT cells (normal keratinocyte cell line) is to compare the cytotoxic effect of phenethyl isothiocyanate.

### Analysis of Morphological Changes by Phase Contrast Microscopy

The morphological changes in PEITC-treated normal and cervical cancer cells were analyzed by phase contrast microscopy as described previously (Kim et al., 2015). 5 × 10^3^ cells were seeded per well in a 96-well plate in triplicate and incubated for 24 h in a 5% CO_2_ incubator at 37°C. Thereafter, the cell lines were re-supplemented with increasing concentrations of PEITC for 24 h, and then cellular morphological changes were observed under a phase contrast microscope and compared with the control untreated cell group.

### Determination of DNA Fragmentation by DAPI Staining

The nuclear morphology of the apoptotic cells was observed by fluorescence microscopy after DAPI staining (Al-Otaibi et al., 2018). Briefly, 5 × 10^3^/ml CaSki and HeLa cells were seeded into a 6-well plate, followed by incubation for 24 h, and then treated with various concentrations of PEITC for 24 h. Thereafter, the cells were washed with cold phosphate-buffered saline (PBS) and fixed in ice-cold 70% methanol for 10 min. The cells were then stained with DAPI and incubated for 15 min at 37°C in the dark. Finally, the cells were again washed with PBS, and the cells were observed under a fluorescence microscope (Nikon ECLIPSE Ti-S, Japan).

### Determination of Apoptosis by Flow Cytometry and Annexin-V FITC/Propidium Iodide Staining

The prominent characteristic of apoptosis is the alteration in the distribution of phosphatidylserine on the two leaflets of the plasma membrane. The apoptosis induction was determined using the annexin V-FITC kit according to the manufacturer’s protocol. In brief, the cells were washed with cold phosphate-buffered saline after 24 h of PEITC treatment and then incubated with 5 µl fluorochrome-conjugated annexin V for 15 min at room temperature. Thereafter, the cell suspension was centrifuged at 600 × g for 5 min and the supernatant was discarded, followed by incubation with 5 µl propidium iodide solution at room temperature for 15 min. Apoptotic cells were immediately analyzed by flow cytometry. Annexin V/PI staining was also performed in accordance with the previously described method (Mannarreddy et al., 2017). Briefly, the cells were treated with various concentrations of PEITC for a period of 24 h and then washed and fixed with methanol and acetic acid (3:1 v/v) for 10 min. The cells were incubated with Annexin V and PI for 30 min at room temperature in the dark and then immediately examined under a fluorescence microscope after washing with PBS.

### Assay of Caspase-3, Caspase-8, and Caspase-9 Activities

Briefly, cells were seeded and allowed to grow for 24 h. The caspase activity in PEITC-treated and untreated cervical cancer cells was determined using caspase colorimetric assay kits. The cells were lysed in 50 µl ice-cold cell lysis buffer and centrifuged for 3 min at 8,000 × g after incubation for 10 min on ice to collect the supernatant. Then, 50 µl cell lysate was transferred into a 96-well plate, and 50 µl of reaction buffer containing 10 mM DTT was added. Thereafter, 5 µl of 4 mM DEVD-pNA substrate was transferred to each well and allowed to incubate for 1 h at 37°C. Finally, the absorbance was measured at 405 nm on a microtiter plate reader.

### Determination of Effect of Caspase Inhibitors

To determine the effect of PEITC, the CaSki and HeLa cells were pretreated with 50 µM each of Z-DEVD-FMK (caspase-3 inhibitor), Z-IETD-FMK (caspase-8 inhibitor), and Z-LEHD-FMK (caspase-9 inhibitor) for 2 h. Then, cells were treated with various concentrations of PEITC for 24 h, and cell survival was determined using MTT assay as described above.

### Determination of Mitochondrial Oxidative Stress

The oxidative stress in mitochondria is measured on the basis of the generation of peroxynitrite through dihydrorhodamine 123 (DHR123), a cell-permeable fluorogenic probe. DHR123 is oxidized into rhodamine 123 upon reacting with peroxynitrite and localizes in the mitochondria, exhibiting green fluorescence (Sobreira et al., 1996). Following PEITC treatment on grown CaSki and HeLa cells for 24 h, 1.25 µM DHR123 was added and incubated for 30 min at 37°C in the dark. Subsequently, the cells were fixed and washed twice with PBS before being examined under the fluorescence microscope.

### Determination of Intracellular ROS Level

The intracellular level of reactive oxygen species was measured using the DCF-DA method as described previously (Wasim and Chopra, 2018). To study the effect of PEITC, CaSki and HeLa cells were seeded into a 12-well plate and incubated for 24 h. The cells were exposed to various concentrations of PEITC for 24 h and then fixed and incubated with 10 µM DCF-DA for 30 min at 37°C, wrapped in aluminum foil. The cells were washed with PBS to remove the excessive DCF-DA, and images were examined under the fluorescence microscope.

### Determination of Effect of an ROS Inhibitor, N-Acetylcysteine

We used an ROS inhibitor, N-acetylcysteine (NAC), to verify the intracellular ROS generation in PEITC-treated CaSki and HeLa cells. Briefly, the cells were incubated with 10 mM NAC for 2 h following the PEITC treatment for 24 h. Thereafter, 10 µM DCFH-DA was added and incubated for 30 min at 37°C in the dark. After washing with PBS, the images were captured using the fluorescence microscope. Additionally, we also checked the cell viability in the presence of NAC using MTT assay as described above.

### Evaluation of Synergistic Interaction Between Phenethyl Isothiocyanate and Cisplatin Using Chou–Talalay’s Method

The effect on cell viability of CaSki cells was determined using MTT cytotoxicity assay after 24 h of incubation, and the combination index (CI) values were calculated using Chou–Talalay’s method **(** Chou and Talalay, 1983; Chou et al., 1994 **)**. The CI for the two drugs at IC_50_ and IC_70_ was calculated using the following equation:

$$\text{CI }=\;{\left(\text{D}\right)}_{1}/{\left(\text{Dx}\right)}_{1}+{\left(\text{D}\right)}_{2}/{\left(\text{Dx}\right)}_{2},$$

where (Dx)_1_ and (Dx)_2_ represent the lone concentrations of PEITC and cisplatin, respectively, which were required for x% inhibition and (D)_1_ and (D)_2_ are the concentrations of PEITC and cisplatin, respectively, used in the combination that together induced x% inhibition. The CI value quantitatively defines synergism (if CI < 1), additive effect (if CI = 1), and antagonism (if CI > 1).

### Statistical Analysis

Statistical analysis was performed using GraphPad Prism 6.0 software. All the experiments presented here were performed in triplicate, and each experiment was repeated three times. The error bars for all the data indicate mean ± standard deviation (SD). The two groups were compared by one- and two-way ANOVA using Dunnett’s and Tukey’s multiple comparison tests, respectively. A *p*-value <0.05 was considered to be significant.

## Results

### Phenethyl Isothiocyanate Exerted Insignificant Cytotoxic Effects on HaCaT Cells

**FIGURE 1:**
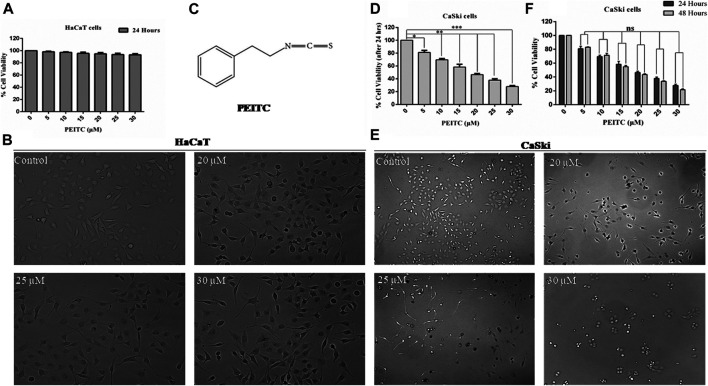
Cell viability assay was performed to assess the effect of PEITC on HaCaT and CaSki cell lines. **(A)** PEITC exerted insignificant cytotoxic effects on HaCaT cells at various doses.** (B)** Morphological analysis showed that phenethyl isothiocyanate at doses of 20, 25, and 30 μM did not significantly alter the morphology of HaCaT 24 h after treatment. **(C)** Represents the structure of phenethyl isothiocyanate. **(D)** Phenethyl isothiocyanate resulted in significant cytotoxic effects in CaSki cells after 24 h of treatment in a dose-dependent manner when compared to the untreated control. **(E)** Cytotoxic effect of phenethyl isothiocyanate as analyzed by phase contrast microscopy. Similar to the cell viability result, phenethyl isothiocyanate sufficiently induced morphological changes in CaSki cells at 20, 25, and 30 μM after 24 h of treatment. **(F)** Inhibition of cell survival after 48 h of treatment with phenethyl isothiocyanate further increased as compared to that after 24 h in CaSki cells. The graphs represent ± SD of three independent experiments. The image is the representation of three independent experiments. *p value < 0.05 compared with the untreated control was considered statistically significant.

The human keratinocytes (normal cell line HaCaT) were used for investigating phenethyl isothiocyanate cytotoxicity through MTT assay (viability) and phase contrast microscopy (morphological changes). As the findings show, exposure of phenethyl isothiocyanate resulted in negligible toxicity to normal cells (HaCaT) up to a dose of 30 μM for 24 h. Percentage of normal cell survival at doses of 20, 25, and 30 μM after 24 h was recorded as 94.78, 93.62, and 93.28%, respectively, which is statistically insignificant compared to the untreated control (Figure 1A). In addition, morphological analysis showed that phenethyl isothiocyanate at doses of 20, 25, and 30 μM did not significantly alter the morphology of HaCaT 24 h after treatment (Figure 1B). Consequently, the findings clearly endorse negligible cytotoxic effects of phenethyl isothiocyanate on normal cells. Figure 1C represents the structure of phenethyl isothiocyanate.

### Phenethyl Isothiocyanate Attenuated Cervical Cancer Cell Proliferation

Similarly, cell viability assay (MTT assay) was performed to assess the anticancerous potential of phenethyl isothiocyanate on CaSki and HeLa cells with various concentrations and incubation times, like 24 and 48 h. Phenethyl isothiocyanate treatment resulted in significant cytotoxic effects (i.e., decreased the cervical cancer cell viability) in a dose- and time-dependent manner when compared to the untreated control. After 24 h of phenethyl isothiocyanate treatment, the inhibition percent recorded for CaSki cells was around 19.08, 30.42, 41.68, 53.52, 62.13, and 72.27% at 5, 10, 15, 20, 25, and 30 µM, respectively, as compared to the untreated control (Figure 1D). Simultaneously, the cytotoxic effect of phenethyl isothiocyanate was also analyzed by phase contrast microscopy. Similar to the cell viability result, phenethyl isothiocyanate has sufficiently induced morphological changes in CaSki cells at 20, 25, and 30 μM after 24 h of treatment (Figure 1E). Moreover, inhibition of cell survival after 48 h of treatment with phenethyl isothiocyanate further increased as compared to that after 24 h (Figure 1F). Moreover, PEITC treatment exerted significant cytotoxicity in HeLa cells in a dose- and time-dependent manner. Thereafter, the morphological changes in PEITC-treated HeLa cells were analyzed by phase contrast microscopy. All the experimental data of HeLa cells are provided in Supplementary Material.

### Cisplatin Attenuated Growth of Normal and Cervical Cancer Cells

**FIGURE 2:**
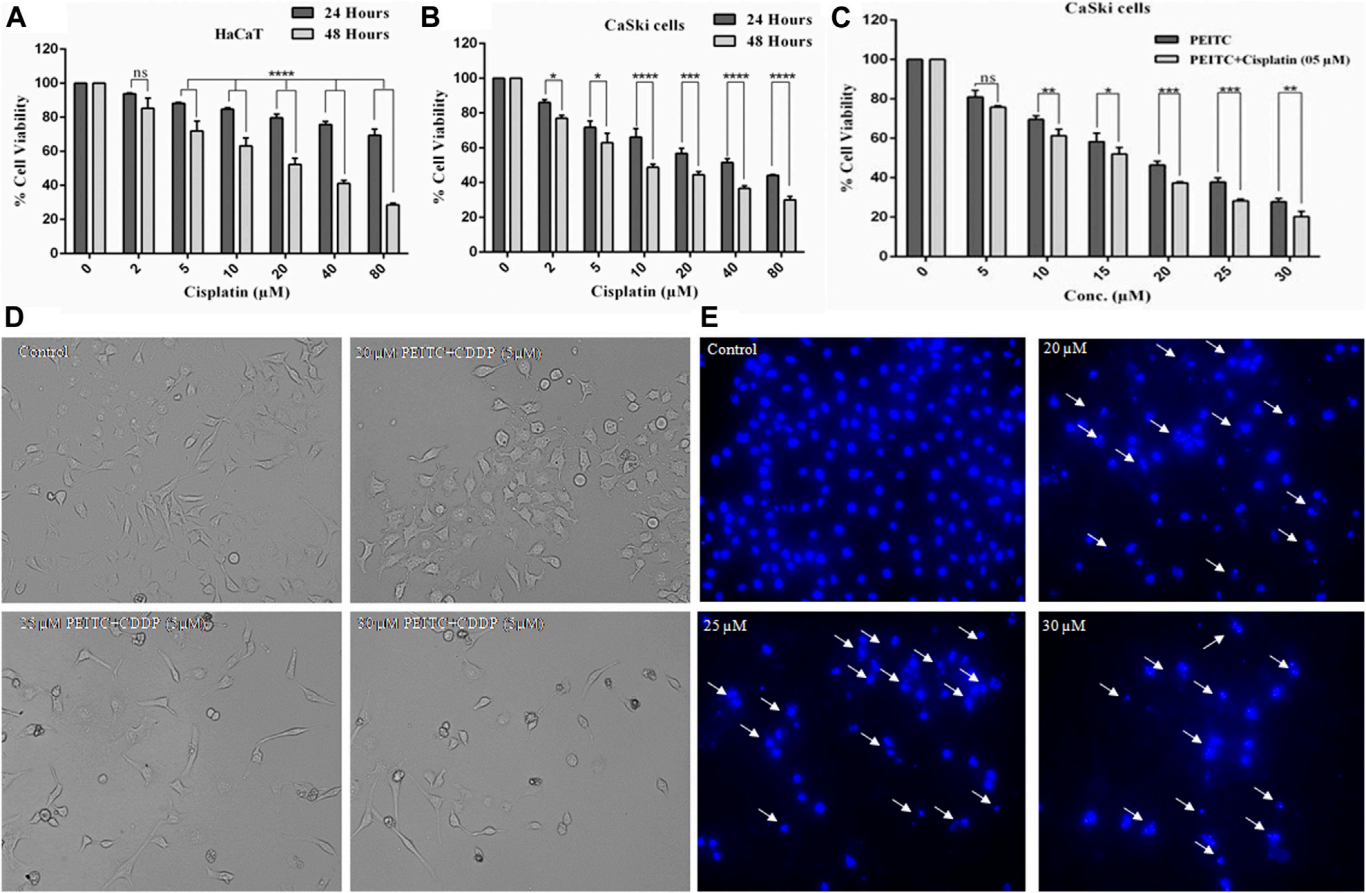
**(A)** Cisplatin exerted cytotoxicity on the normal cell line (HaCaT).** (B)** Cisplatin-treated CaSki cells showed significant reduction in cell viability in a dose and time dependent manner.** (C)** Percent cell viability of CaSki cells exposed to various doses of PEITC (5-30 μM) accompanied with 5 μM cisplatin compared to individual PEITC-treatment (5-30 μM). **(D)** Combined doses of PEITC with sub-optimal concentration of cisplatin (5 μM) exerted significant cytotoxic effects on CaSki cells. **(E)** CaSki cells were incubated with different concentration of PEITC (20, 25 and 30 μM) for 48 h, then nuclear condensation and fragmentation (white arrows) was detected by fluorescence microscopy. The data represents mean ±SD of three independent experiments. The image is the representation of three independent experiments. *p value < 0.05 compared with the untreated control was considered statistically significant.

To gain an understanding of the comparative cytotoxicity, cancer cell lines (CaSki and HeLa) and normal cells were treated with different doses of cisplatin over 24 and 48 h. The results of cell viability assay showed that cervical cancer cells and normal cells are similarly susceptible to cisplatin. After 48 h of cisplatin treatment, HaCaT cells showed 85.29, 71.95, 63.20, 52.25, 41.14, and 28.61% survival at the corresponding doses of 2, 5, 10, 20, 40, and 80 µM, respectively (Figure 2A). Briefly, after 24 h of treatment, the cell survival percent of CaSki cells at various doses of 2, 5, 10, 20, 40, and 80 µM was found to be 86, 71.75, 66.11, 56.81, 51.52, and 44.09%, respectively, as compared to the untreated control. On the other hand, after 48 h of exposure, the cell viability of cisplatin-treated CaSki cells was further reduced but not very significantly compared to that after 24 h, which was observed as 76.94, 62.85, 48.75, 44.43, 36.59, and 30.03% at the same doses of 2, 5, 10, 20, 40, and 80 µM, respectively (Figure 2B). Furthermore, cisplatin-treated HeLa cells also showed cytotoxicity in a dose- and time-dependent manner (Supplementary Material).

### Phenethyl Isothiocyanate Exerted Synergistic Effects With Cisplatin on CaSki Cells

Growth inhibitory effects of phenethyl isothiocyanate on the cervical cancer cells triggered a great interest in investigating the combined effects of phenethyl isothiocyanate with cisplatin on CaSki cells. The cervical cancer cells were grown and treated with a suboptimal dose of cisplatin (5 µM) combined with increasing doses of phenethyl isothiocyanate (5–30 µM), and cell viability was estimated using MTT assay. After 24 h of incubation, the result of a substantial decrease in proliferation, which was 24.21, 38.66, 48.02, 62.59, 71.81, and 79.9% at the concentrations of 2, 5, 10, 20, 40, and 80 μM of phenethyl isothiocyanate, respectively, in combination with 5 μM cisplatin, compared to phenethyl isothiocyanate alone, suggested that phenethyl isothiocyanate inhibits cell survival synergistically when combined with a suboptimal dose of cisplatin (Figure 2C). The combination index (CI) method of Chou–Talalay was used to ascertain the synergistic interaction between PEITC and cisplatin. The CI values at IC_50_ and IC_70_ were found to be 0.875 and 0.892, respectively. Both the values being less than one clearly shows the synergism between PEITC and cisplatin in inhibiting the growth of cancer cells. Moreover, morphological changes were also observed in cervical cancer cells exposed to phenethyl isothiocyanate combined with a suboptimal dose of cisplatin (5 μM) (Figure 2D). Similarly, HeLa cells were also treated with various doses of PEITC combined with a suboptimal dose of cisplatin (5 μM), followed by morphological analysis (Supplementary Material).

### Phenethyl Isothiocyanate Induced Nuclear Fragmentation and Condensation in Cervical Cancer Cells

Mostly, apoptosis involvement is delineated through the assessment of prominent nuclear fragmentation and condensation. DAPI staining was performed to confirm whether the proliferation inhibition of phenethyl isothiocyanate–treated cervical cancer cells was due to apoptosis. The results indicated that phenethyl isothiocyanate significantly induced nuclear changes at various doses (20, 25, and 30 μM) post treatment for 24 h. Phenethyl isothiocyanate induced nuclear fragmentation and condensation in cervical cancer cells in a dose-dependent manner, as indicated in the photomicrographs (Figure 2E). Thus, the results suggested that phenethyl isothiocyanate possesses apoptotic potential to attenuate the cervical cancer cell proliferation.

### Phenethyl Isothiocyanate Induced Apoptosis in the Cervical Cancer Cells

**FIGURE 3:**
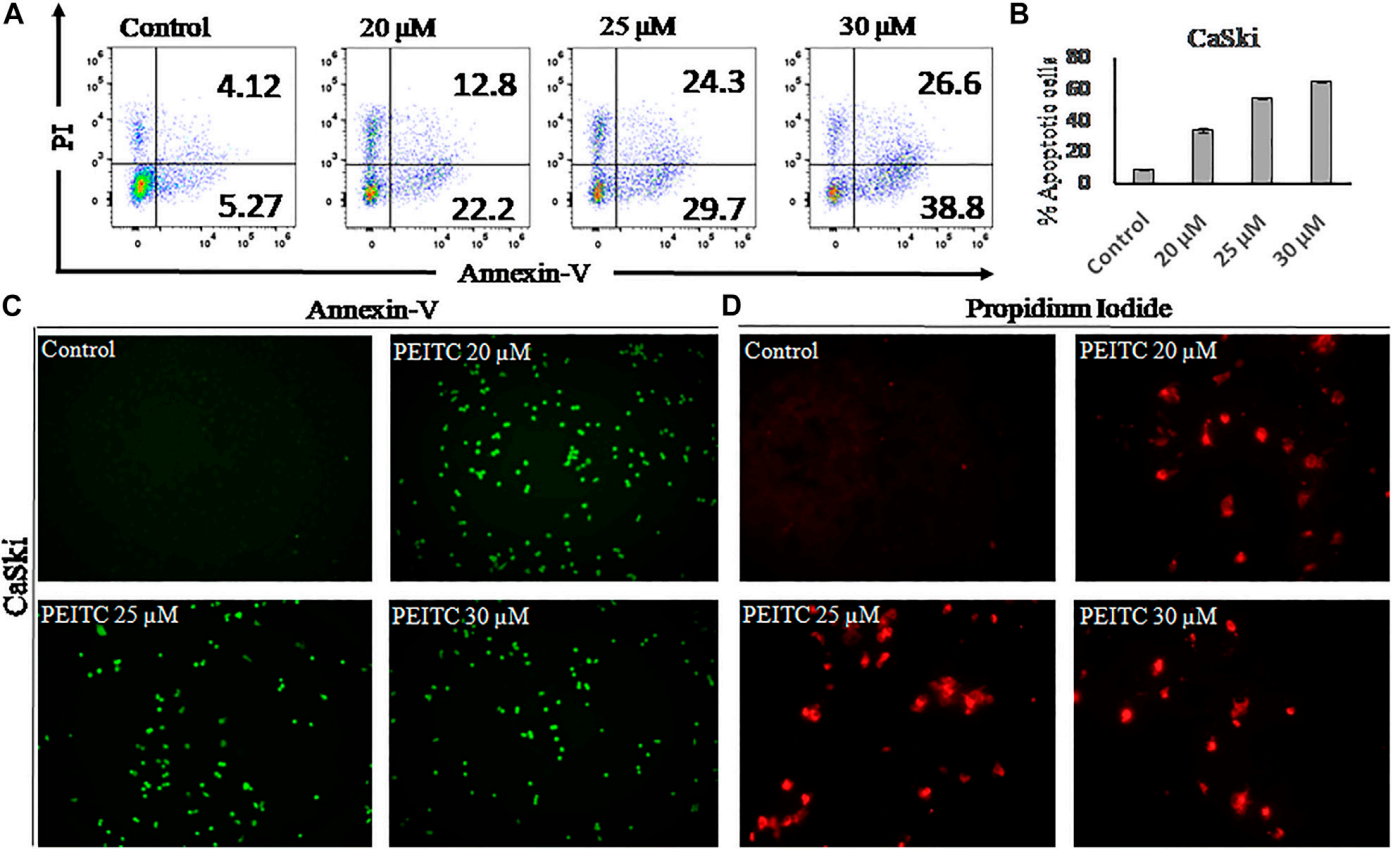
**(A)** After 24 hours apoptosis induction was quantified in PEITC-treated CaSki cells by Annexin V-FITC kit using flow cytometry. **(B)** Apoptosis percentage in PEITC-treated CaSki cells was measured. CaSki cells were induced with different concentration of PEITC (20, 25 and 30 μM) for 24 h and fluorescent microscopic images of Annexin V-FITC/PI stained cells were captured to examine the induction of apoptosis in CaSki cells under fluorescent microscope. PI can bind with damaged nucleus of cancer cells and Annexin V-FITC binds with phosphatidylserine of the plasma membrane. (**C** **and** **D**) Control untreated CaSki cells appear non-fluorescent while the fluorescent bright green and red demonstrate induction of apoptosis in CaSki cells. The data represents mean ±SD of three independent experiments. The image is the representation of three independent experiments.

Apoptosis is observed *via* the movement of phosphatidylserine from the inner leaflet to the outer leaflet of the plasma membrane. In order to check the apoptotic effect of phenethyl isothiocyanate on CaSki cells, we used the phosphatidylserine-specific membrane-impermeable dye annexin-V with the FITC tag and the nuclear staining dye PI. Therefore, to elaborate on the effect of PEITC on cervical cancer, CaSki and HeLa cells were treated with various doses of PEITC for 24 h, and then the cells were trypsinized and harvested for flow cytometry, pronouncing the apoptosis induction in cervical cancer cells (Figures 3A,B). The staining results of CaSki and HeLa cells (green fluorescence) showed substantial annexin V-FITC binding and showed a considerable apoptosis induction in the phenethyl isothiocyanate–treated cancer cells compared to the untreated control (Figure 3C). Furthermore, the red fluorescence observed in phenethyl isothiocyanate–administered CaSki and HeLa cells stipulates the PI stain of the nucleus in the apoptotic cells (Figure 3D).

### Phenethyl Isothiocyanate Caused Activation of Caspases (Caspase-8, -9, and -3) in Cervical Cancer Cells

**FIGURE 4:**
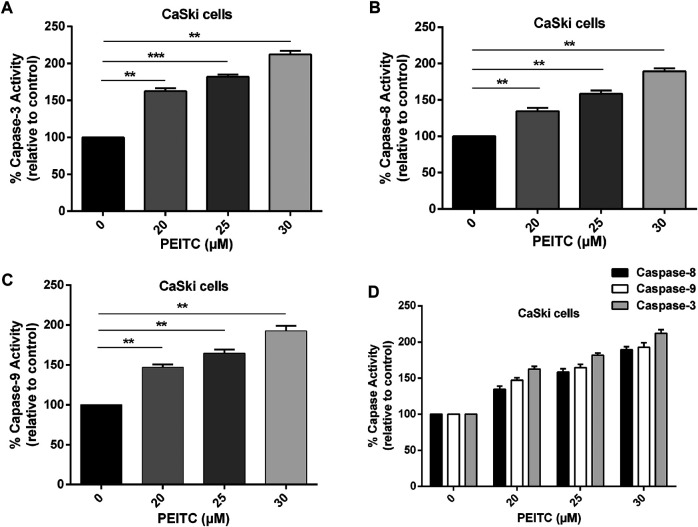
Effect of PEITC on apoptosis induction as assessed by caspase-3, -8 and -9 activity assays. **(A-C)** PEITC-treatment (20, 25 and 30 μM) resulted in induction of apoptosis in CaSki cells through activation of caspase-3, -8 and -9 after 24 h. **(D)** Combined graph exhibiting activation of various cellular caspases in cervical cancer cells after PEITC-treatment. The data represents mean ±SD of three independent experiments. *p value < 0.05 compared with the untreated control was considered statistically significant.

The role of caspases in phenethyl isothiocyanate–mediated apoptosis cells was investigated to gain insight at the molecular level. The caspases belong to the family of cystein proteases which exist as inactive zymogens in the cells. One of the key factors at the onset of apoptosis is a cascade of catalytic activation of caspases, which further leads to apoptosis. Particularly, caspase-8 and caspase-9 are known as initiator caspases of the extrinsic and intrinsic apoptosis pathways, respectively, while caspase-3 is the main executioner of apoptosis. So, we measured caspase activity in phenethyl isothiocyanate–treated and untreated cervical cancer cells. Phenethyl isothiocyanate–treated CaSki cells showed significant caspase activity in a concentration-dependent manner. The results indicated that phenethyl isothiocyanate administration caused increased caspase-3 activities in cervical cancer cells as 62.53, 81.89, and 112.06% at 20, 25, and 30 µM, respectively, as compared to the untreated control (Figure 4A). Likewise, a substantial dose-dependent increment in caspase-8 and caspase-9 activities was observed in cervical cancer cells at the same doses as depicted in Figures 4B–D.

### Caspase Inhibitors Attenuated Phenethyl Isothiocyanate–Mediated Apoptosis in Cervical Cancer Cells

**FIGURE 5:**
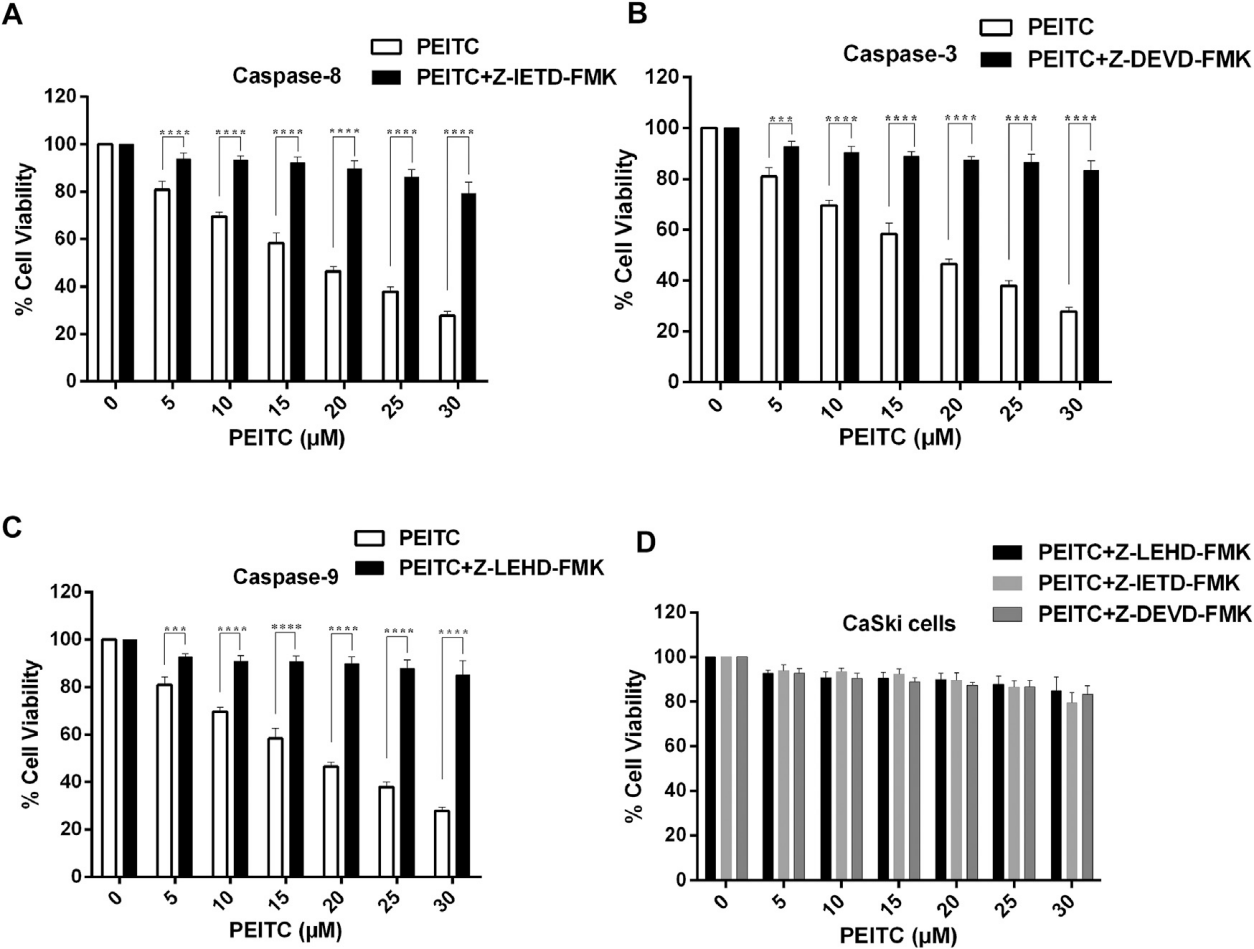
Effect of PEITC on activation of caspases in CaSki cells as assessed in the presence of caspase inhibitors. Percent cell viability of PEITC-treated (20, 25 and 30 μM) CaSki cell was determined in the presence of caspase inhibitors by MTT assay. **(A)** Z-DEVD-FMK-caspase-3 inhibitor, **(B)** Z-IETD-FMK-caspase-8 inhibitor and **(C)** Z-LEHD-FMK-caspase-9 inhibitor. **(D)** Combined graph showing percent cell viability of PEITC-treated CaSki cells in the presence of caspase inhibitors. The data represents mean ±SD of three independent experiments. *p value < 0.05 compared with the untreated control was considered statistically significant.

We further studied the cell viability assay of the phenethyl isothiocyanate–mediated cytotoxic effect of CaSki and HeLa cells in order to understand the role of the caspase inhibitors (Z-DEVD-FMK, Z-IETD-FMK, and Z-LEHD-FMK for caspase-3, -8, and -9, respectively). We observed significant reduction in the inhibition percent of cell survival in phenethyl isothiocyanate–exposed cervical cancer cells which were pretreated with caspase inhibitors, as shown in Figures 5A–D. Phenethyl isothiocyanate mediated apoptosis due to caspase activation in cervical cancer cells, simultaneously; caspase inhibitors did not fully mitigate phenethyl isothiocyanate–induced cytotoxicity, indicating an additional function of the caspase-independent pathway.

### Phenethyl Isothiocyanate Induced ROS Generation in Mitochondria of Cervical Cancer Cells

**FIGURE 6:**
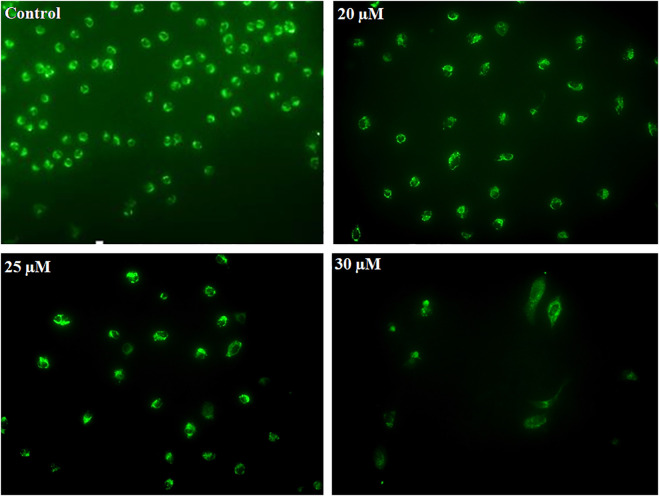
CaSki cells were exposed to different concentration of PEITC (20, 25 and 30 μM) for 24 h. Then, cervical cancer cells were analyzed for mitochondrial ROS generation by fluorescence microscope using DHR123. The image is the representation of three independent experiments.

Early apoptosis is marked by the decreased mitochondrial membrane potential. Dihydrorhodamine 123 is a non-fluorescent molecule until it oxidizes to fluorescent rhodamine 123 in the presence of peroxynitrite, which is localized in the mitochondria and exhibits green fluorescence. After PEITC treatment, CaSki and HeLa cells were analyzed to determine the mitochondrial ROS generation. PEITC-treated cells were analyzed for peroxynitrite using a fluorescence microscope. PEITC-treated cervical cancer cells showed oxidation of dihydrorhodamine 123, which indicates the production of mitochondrial reactive oxygen species, particularly peroxynitrite. Decreased green fluorescence suggested that PEITC significantly exerted cytotoxic effects with increased concentration as cells undergo apoptosis (Figure 6).

### Phenethyl Isothiocyanate Elevated Intracellular ROS Production in Cervical Cancer Cells

**FIGURE 7:**
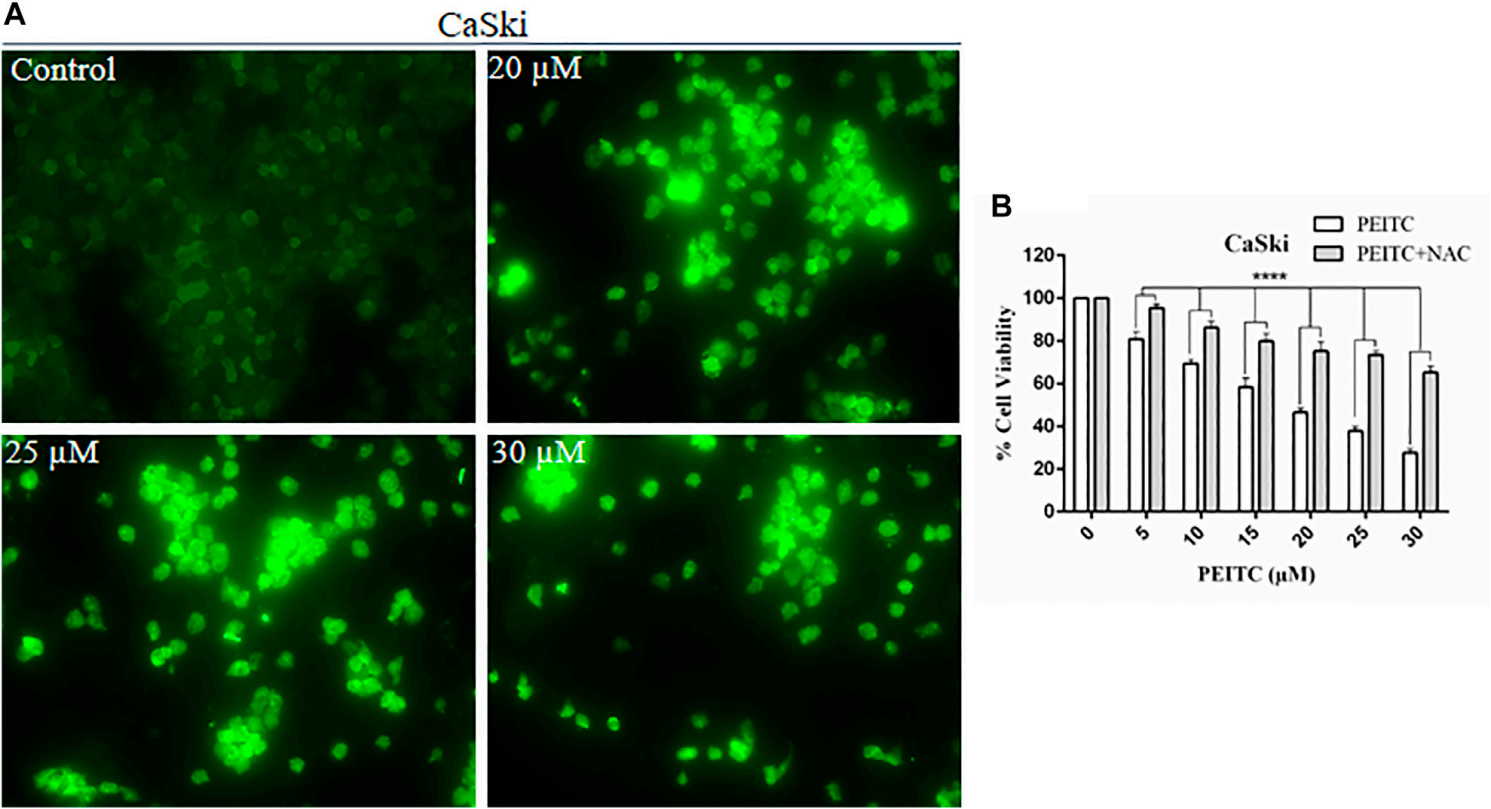
Analysis of ROS generation using DC-FDA staining on CaSki cells treated with PEITC for 24 h and the images were captured by fluorescence microscopy. **(A)** PEITC treatment resulted in significant production of ROS in cervical cancer cells. **(B)** Percent cell viability of PEITC treated (20, 25 and 30 μM) CaSki cells was assessed in the presence of ROS inhibitor, NAC, by MTT assay. The data represents mean ±SD of three independent experiments. *p value < 0.05 compared with the untreated control was considered statistically significant.

The cell death may be caspase-dependent or -independent, which is validated through intracellular reactive oxygen species production (ROS). Therefore, to explore the mechanism of action, we further investigated the ROS level in phenethyl isothiocyanate–treated and untreated CaSki and HeLa cells. Strong DCF-fluorescence in phenethyl isothiocyanate–treated CaSki cells showed an elevated level of ROS generation, as compared to the untreated control. Furthermore, the results of DCF-fluorescence also indicated that phenethyl isothiocyanate enhanced ROS generation in cervical cancer cells in a dose-dependent manner (20, 25, and 30 µM) (Figure 7A).

### N-Acetylcysteine Suppressed Phenethyl Isothiocyanate–Mediated Cytotoxicity in Cervical Cancer Cells

Furthermore, to understand the effect of the ROS inhibitor N-acetylcysteine (NAC) on phenethyl isothiocyanate–mediated cytotoxic effects over CaSki and HeLa cells, we executed cell viability assay at different doses of PEITC. As discussed in the methodology section, cancer cells were treated with 10 mM NAC for 2 h, prior to phenethyl isothiocyanate treatment. The results of viability of NAC-pretreated cells at various doses of phenethyl isothiocyanate (5, 10, 15, 20, 25, and 30 µM) were observed to be 95.45, 86.27, 79.89, 75.20, 73.56, and 65.24%, which was comparatively more than that of the cells not treated with the ROS inhibitor, as depicted in Figure 7B. Thus, the results advised that intracellular ROS generation plays a crucial role in phenethyl isothiocyanate–mediated apoptosis in cervical cancer cells. At the same time, we also observed that the existence of an ROS inhibitor (NAC) was not fully successful in inhibiting phenethyl isothiocyanate cytotoxicity in CaSki and HeLa cells, suggesting the possibility of ROS-independent apoptosis pathways in phenethyl isothiocyanate–treated cervical cancer cells.

## Discussion

At present, the most considerable treatment strategies for cervical cancer are conventional approaches, which include chemotherapy, radiotherapy, and surgery. However, each of the conventional treatment strategies is associated with various complications, such as drug resistance, adverse side effects, low drug efficacy, and cancer recurrence. Formerly, several naturally occurring chemopreventive and chemotherapeutic plant products had been recognized; these trigger a great interest in searching for novel plant products which were reported to have high efficacy, target specificity, and low side effects.

Several recent studies have shown that isothiocyanates obtained from cruciferous plants can be effective in reducing the risk of many cancers as they can cause chemopreventive effects by involving various signaling pathways (Popolo et al., 2017; Abbaoui et al., 2018; Pan et al., 2018; Zhang et al., 2018).Previously, the effect of phenethyl isothiocyanate on KB and HEp-2 indicated growth inhibition and apoptosis induction, exhibited through death receptors 4 and 5(Huong et al., 2011). In the present study, we coherently sought to elucidate the effects of phenethyl isothiocyanate in combination with cisplatin on cervical cancer cells. The aim of the study is to minimize adverse effects and increase medication tolerance and efficacy of traditional standard drugs. It was found that when cervical cancer cells were treated with phenethyl isothiocyanate alone, the viability of cancer cells was significantly reduced in a dose-dependent manner, but in combination with a suboptimal concentration of cisplatin, the viability of cancer cells was further reduced. Phenethyl isothiocyanate and cisplatin together have more cytotoxicity effects than phenethyl isothiocyanate or cisplatin separately, and this indicates that there is an enhanced sensitization of phenethyl isothiocyanate when given in conjunction with the suboptimal dose of cisplatin. The results ascertain that there is synergy between phenethyl isothiocyanate and cisplatin and together they can be considered as a healthy chemical prevention combination in the management of cervical cancer. In conjunction with cell viability results, treatment with phenethyl isothiocyanate, or combination with cisplatin, the morphological examination has also advocated distortion, shrinkage, and detachment of the cervical cancer cells. Earlier *in vitro* experiments also showed the similar effect of phenethyl isothiocyanate on multiple cancer cell lines (Huong et al., 2012; Chen et al., 2013; Ma et al., 2017). On the contrary, the typical cytotoxicity of human keratinocytes does not display phenethyl isothiocyanate, as cisplatin therapy resulted in the cytotoxicity of the normal human keratinocytes.

Chemopreventive agents are capable of regulating the accelerated growth of cancer cells through activation of apoptosis (Braicu et al., 2020). Apoptosis induction can be marked by the fragmentation and condensation of the cell nucleus, inside and outside the membrane leaflets (Nagata, 2000; Saraste and Pulkki, 2000; Balasubramanian et al., 2007). To elucidate the plausible apoptotic mode of action of phenethyl isothiocyanate, nuclear staining and binding of FITC-annexin V with exposed phosphatidylserine were executed. The DAPI staining result indicated condensation and fragmentation of the nucleus, which suggest the induction of the apoptotic cell death in cervical cancer cells when grown in the presence of phenethyl isothiocyanate and cisplatin. The findings of the FITC-annexine V binding were subsequently shown to induce apoptosis in cervical cancer cells with the use of phenethyl isothiocyanate and cisplatin.

Cystein proteases are known to perform multifaceted functions, including apoptosis (Estaquier et al., 2012). Usually, a cascade of caspase activation takes place during apoptosis induction (Degterev et al., 2003). Caspases are synthesized as inactive zymogens (Stennicke et al., 2000), and their activation occurs in response to a particular death signal (Green and Llambi, 2015). The results of caspase activation suggested the involvement of caspase-3, caspase-9, and caspase-8 in apoptosis induction, and the considerable reduction of the cytotoxicity of phenethyl isothiocyanate in the presence of the caspase inhibitor confirmed the caspase-3 participation in execution of the apoptosis in phenethyl isothiocyanate–administered cervical cancer cells. The change in mitochondrial membrane potential during apoptosis is linked to mitochondrial disruption, which may lead to cytochrome c releasing into the cytosol (Gottlieb et al., 2003; Troiano et al., 2007). It has previously been shown that the plant extract has an anticancer activity by apoptosis, which was regulated by the modulation of the mitochondrial membrane potential mechanism and/or by the caspase-3–dependent pathway (Denning et al., 2002; Mantena et al., 2006; Lin et al., 2016; Jaudan et al., 2018). The results of our studies also showed that phenethyl isothiocyanate perturbed mitochondrial functioning, which suggested the involvement of cytochrome-c–dependent apoptosis in cervical cancer cells.

In different biological systems, free radicals are formed and persistently removed, such as reactive oxygen and reactive nitrogen species, and they have been known to perform many critical roles (Pelicano et al., 2004). In cancer cells, the excessive production of ROS may be accompanied by oxidation of macromolecules including proteins, lipids, and nucleic acids. ROS production can be amplified by chemotherapeutic compounds to kill the cancer cells through ROS-mediated apoptosis, conferring the modulation of the redox status in cancer cells (Schumacker et al., 2006; Trachootham et al., 2009). We found that phenethyl isothiocyanate augmented intracellular ROS production in cervical cancer cells, which further triggered nucleus condensation and fragmentation, mitochondrial disruption, and apoptosis induction. Furthermore, phenethyl isothiocyanate–mediated amelioration of intracellular ROS generation was attenuated when cervical cancer cells were pretreated with NAC (ROS inhibitor), which further justifies the augmented ROS level as being due to cytotoxicity of phenethyl isothiocyanate. Therefore, it can be stated that plausibly phenethyl isothiocyanate alone and in combination with cisplatin could induce apoptosis in cervical cancer cells through the disruption of mitochondria and caspase activation by intracellular ROS generation.

## Conclusion

Plant products have been used as chemotherapeutic and chemopreventive agents in various infectious and noninfectious diseases for decades. Several phytochemicals have been identified that have anti-inflammatory, antioxidant, and anticancer properties. The findings of this study have provided insights into the effects of phenethyl isothiocyanate on cervical cancer cells, either on its own or in combination with cisplatin, as can be seen from the growth inhibitions, mitochondrial damage, increased intracellular ROS development, and stimulation of caspases in apoptosis induction. Altogether, in the present study, we have observed that phenethyl isothiocyanate is an excellent compound for the management of cervical cancer. Therefore, we can state that employing phenethyl isothiocyanate could help in cervical cancer management if used in combination with the standard cancer drugs.

## Data Availability

The original contributions presented in the study are included in the article/Supplementary Material; further inquiries can be directed to the corresponding author.
