# Supplementary material for: Phenethyl Isothiocyanate Induces Apoptosis Through ROS Generation and Caspase-3 Activation in Cervical Cancer Cells
Source: Front Pharmacol. 2021 Jul 29;12:673103. doi: 10.3389/fphar.2021.673103 (PMC8358204; doi:10.3389/fphar.2021.673103)
Supplement: Supplementary file 1 [file DataSheet1.docx]

**Phenethyl isothiocyanate induces apoptosis through ROS generation and caspase-3 activation in cervical cancer cells**

*Shoaib^1^, Saba Tufail^1^, Mohammad Asif Sherwani^2^, Nabiha Yusuf^2^, Najmul Islam^1*^*

*^1^Department of Biochemistry, J.N.M.C, Aligarh Muslim University, Aligarh*

*^2^Department of Dermatology, University of Alabama at Birmingham, Alabama, USA.*

***^*^Email****:* [*najmulamu@gmail.com*](mailto:najmulamu@gmail.com)

**SUPPLEMENTARY MATERIAL**


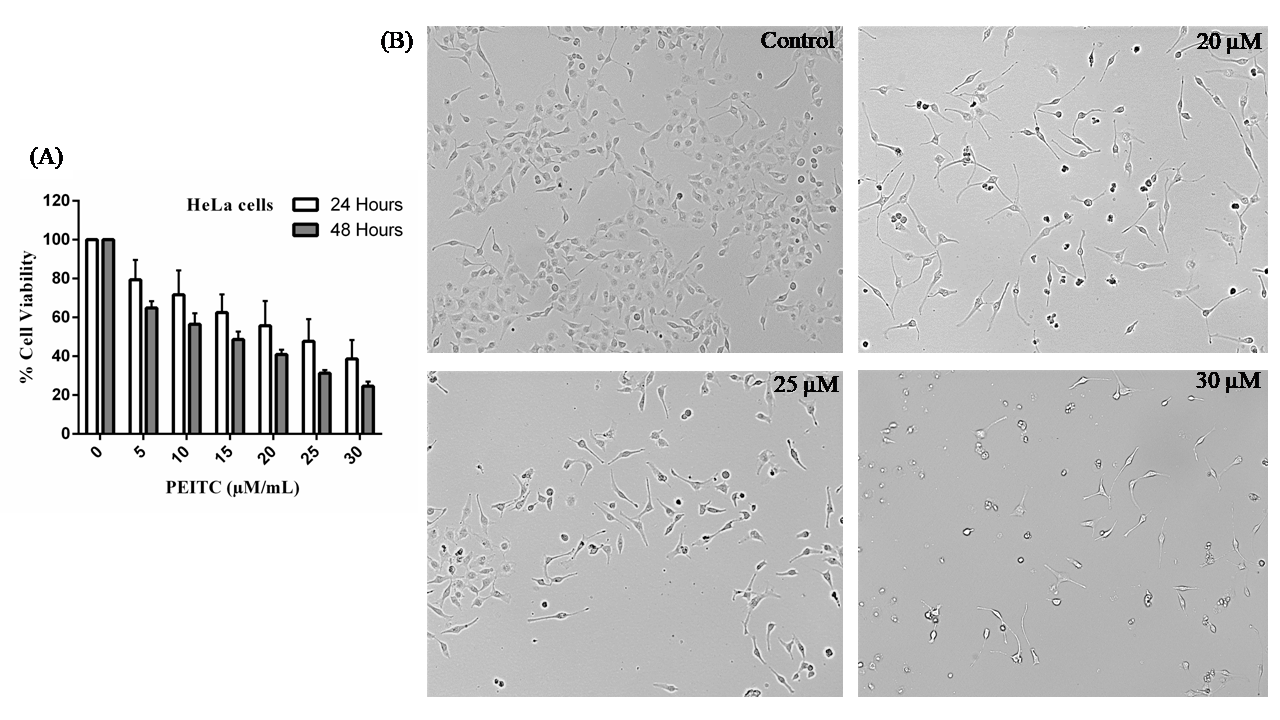
**Figure S1.**(A) PEITC exerted significant cytotoxicity in HeLa cells in a dose and time dependent manner. (B) The phase contrast microscopy revealed distorted morphology of PEITC-treated HeLa cells after 24 hours. The graphs indicate mean ±SD of three independent experiments. The image is the representation of three independent experiments.


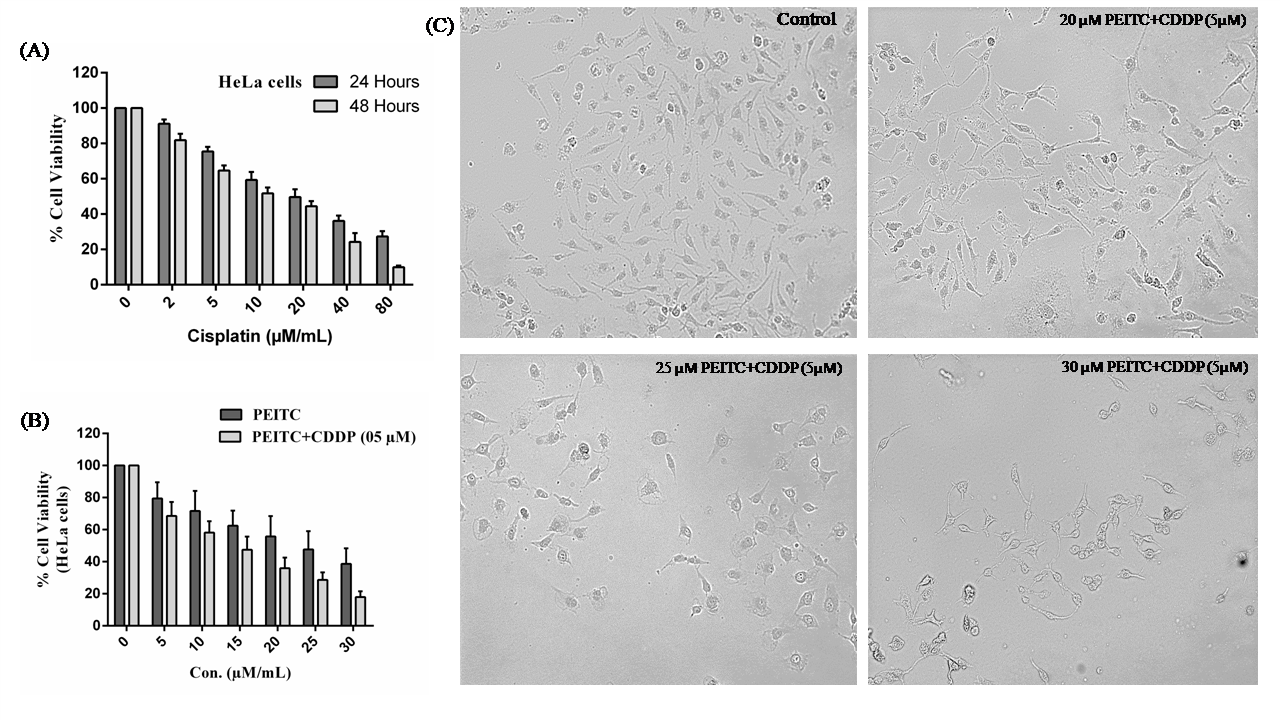


**Figure S2.**The cytotoxic effects cisplatin (CDDP) were assessed on HeLa cells by MTT assay.(A) Cisplatin showed significant reduction in cell viability after 24 and 48 hours. (B) Thereafter, the percent viability was significantly reduced when HeLa cells were treated with combined doses of PEITC and sub-optimal cisplatin concentration (05μM). (C) Similarly, HeLa cells showed distorted morphology when treated with PEITC+cisplatin (5 μM). The graphs indicate mean ±SD of three independent experiments. The image is the representation of three independent experiments.


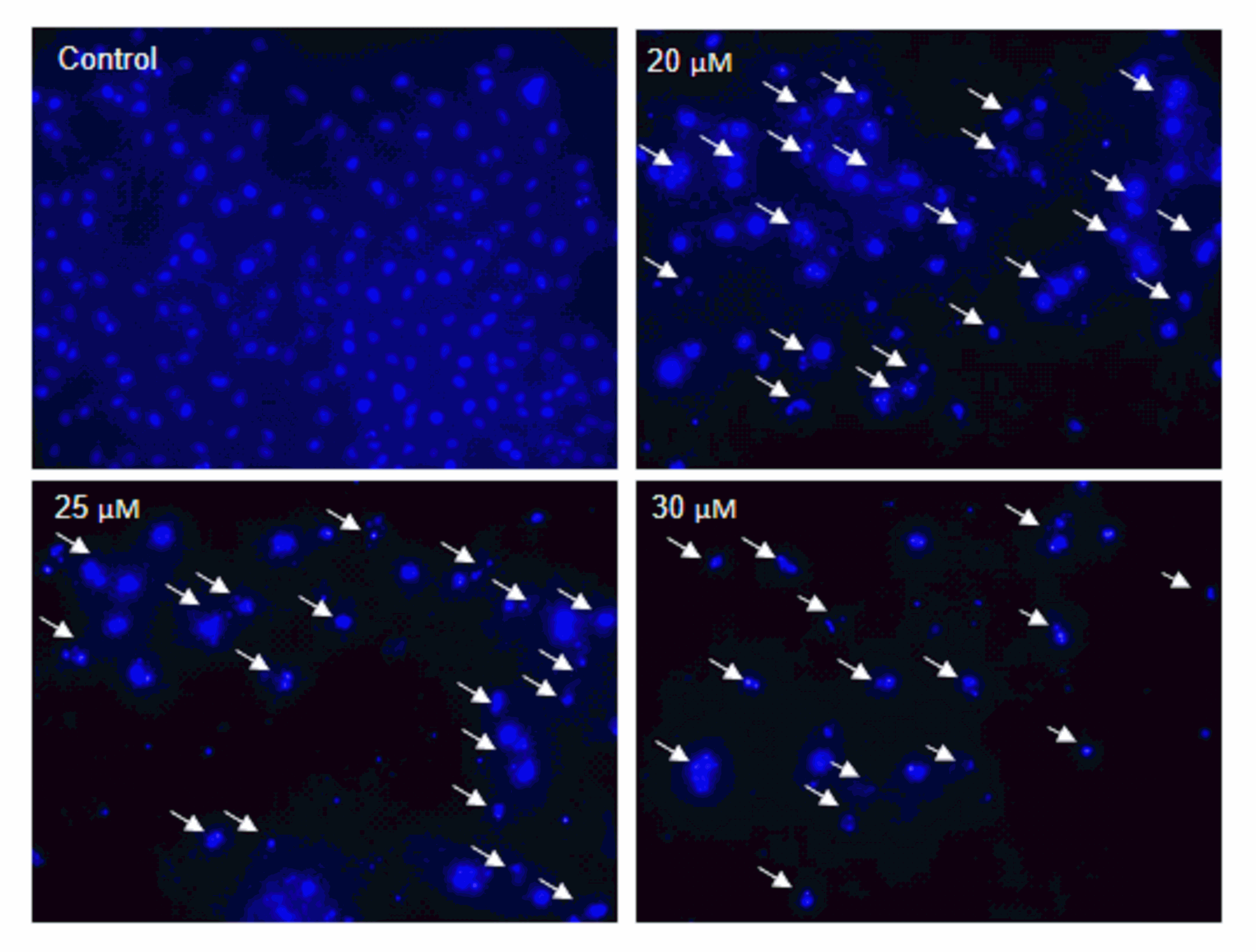


**Figure S3.** The apoptosis induction in PEITC-treated HeLa cells was checked by DAPI staining. Fluorescence microscopy images indicate nuclear condensation and fragmentation (white arrows) after incubating HeLa cells with PEITC for 48 hours. The image is the representation of three independent experiments.


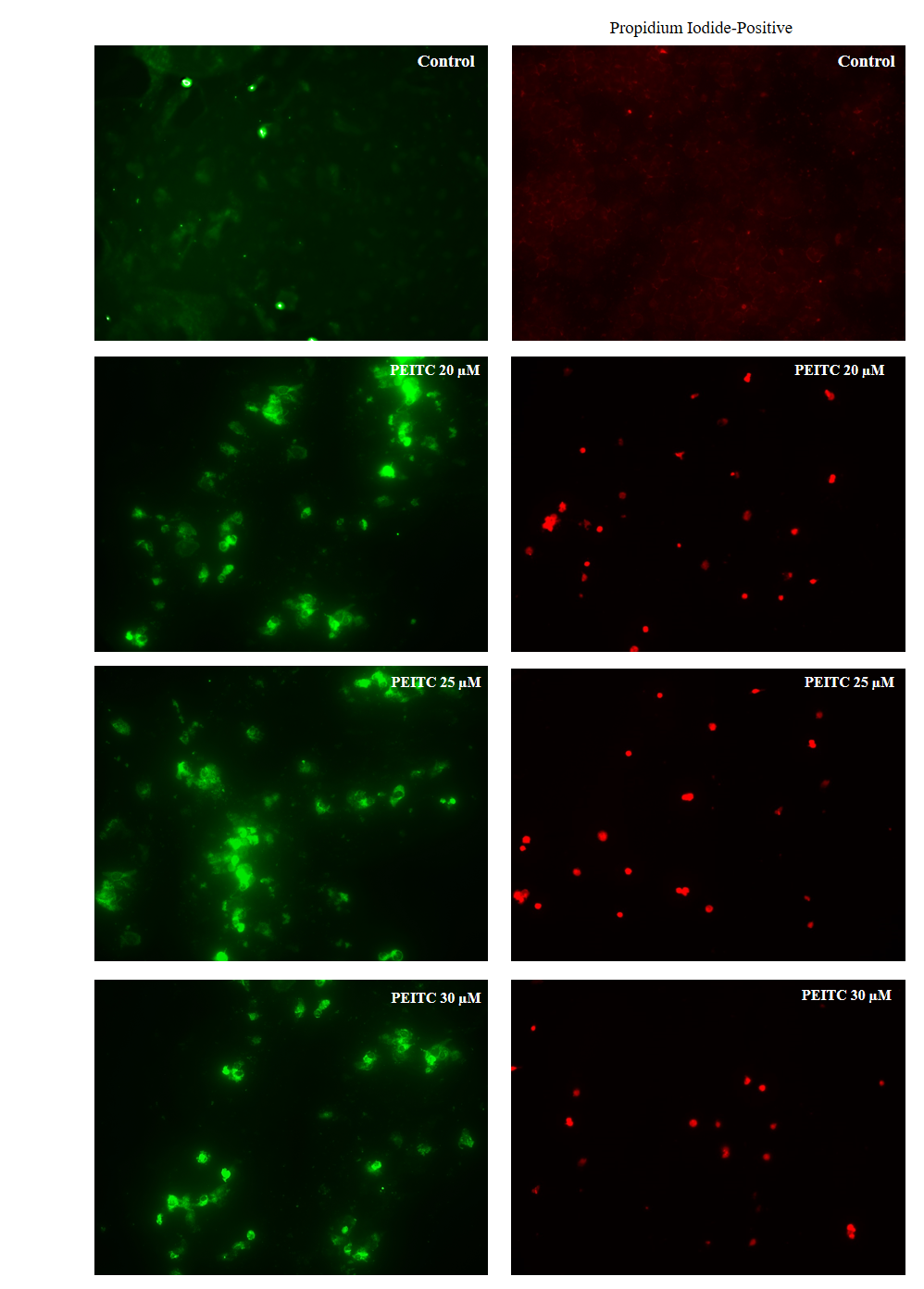


**Figure S4.** HeLa cells were induced with different concentration of PEITC (20, 25 and 30 μM) for 24 h and fluorescent microscopy images of Annexin V-FITC/PI stained cells were captured to examine the induction of apoptosis in HeLa cells under fluorescent microscope. PI can bind with damaged nucleus of cancer cells and Annexin V-FITC binds with phosphatidylserine of the plasma membrane. Control untreated HeLa cells appear non-fluorescent while the fluorescent bright green and red demonstrate induction of apoptosis in HeLa cells. The image is the representation of three independent experiments.


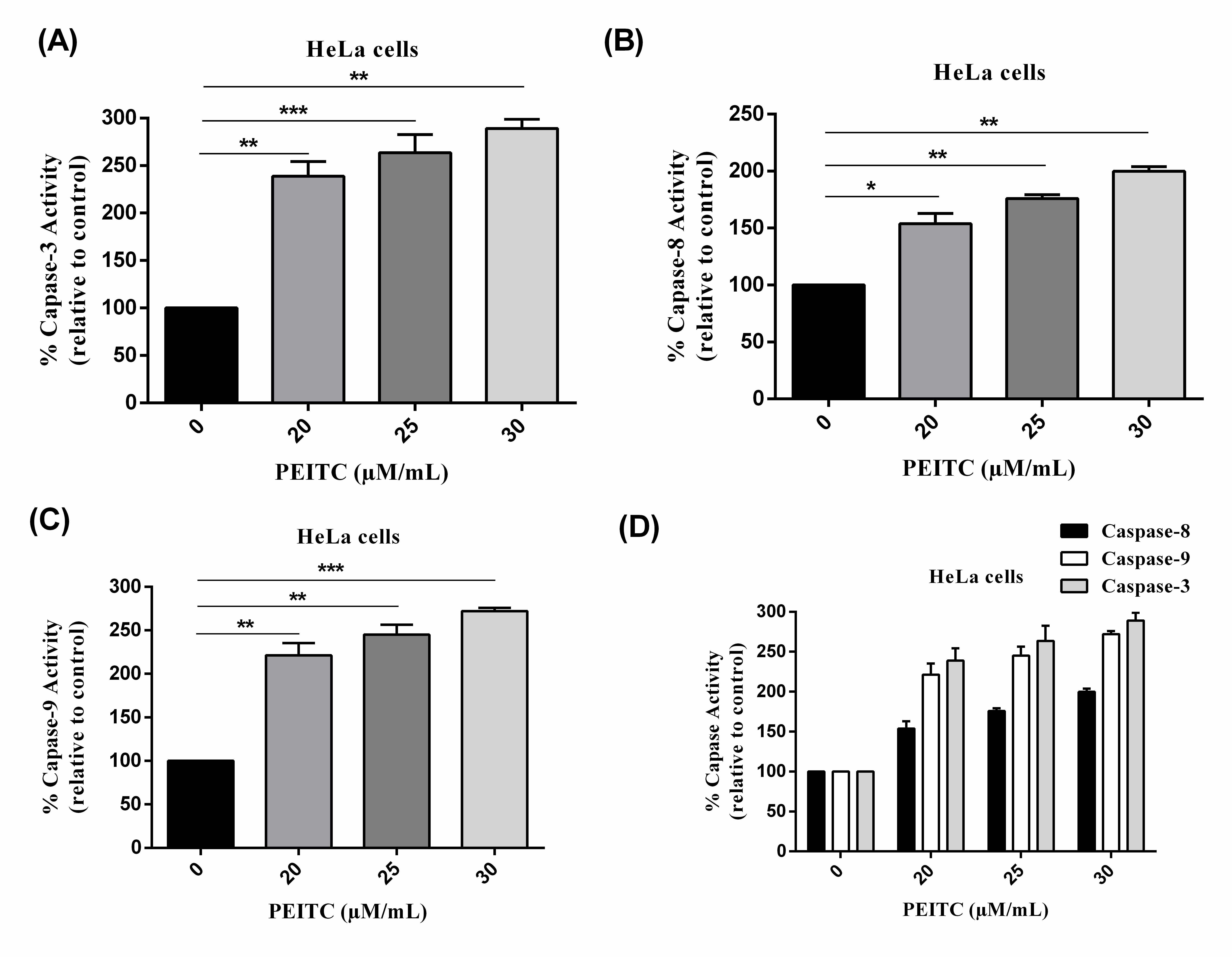


**Figure S5.** Effect of PEITC on apoptosis induction was assessed by caspase-3, -8 and -9 activity assays. As shown in the figure PEITC-treatment (20, 25 and 30 μM) resulted in induction of apoptosis in HeLa cells through activation of caspase-3, -8 and -9 after 24 h. The data represents mean ±SD of three independent experiments. *p value < 0.05 compared with the untreated control was considered statistically significant.


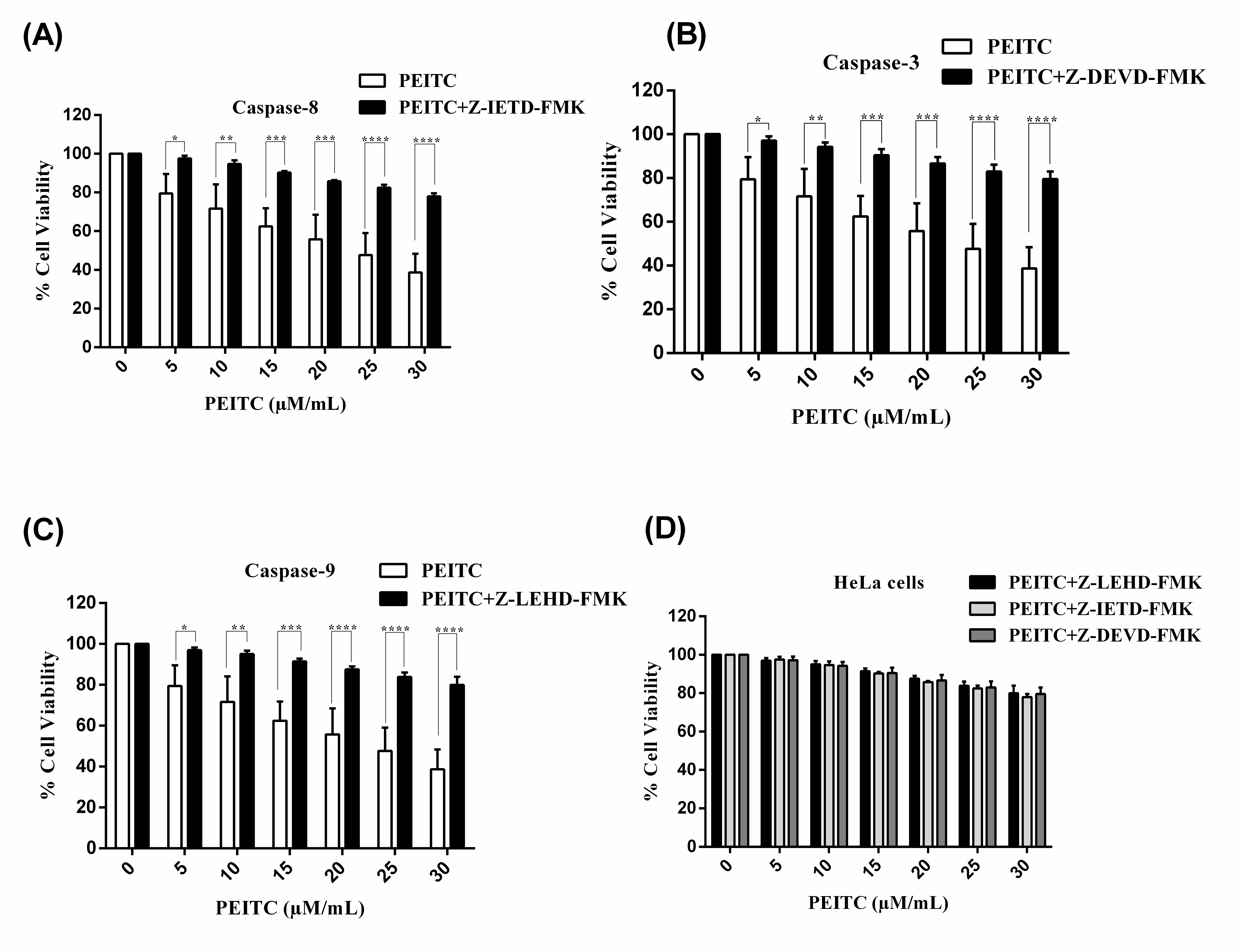


**Figure S6.** Effect of PEITC on activation of caspases in HeLa cells was assessed in presence of caspase inhibitors (Z-DEVD-FMK-caspase-3 inhibitor, Z-IETD-FMK-caspase-8 inhibitor and Z-LEHD-FMK-caspase-9 inhibitor). As shown in the figures, percent cell viability of PEITC-treated HeLa cells (20, 25 and 30 μM) was assessed in presence of caspase inhibitors by MTT assay. The data represents mean ±SD of three independent experiments. *p value < 0.05 compared with the untreated control was considered statistically significant.

**
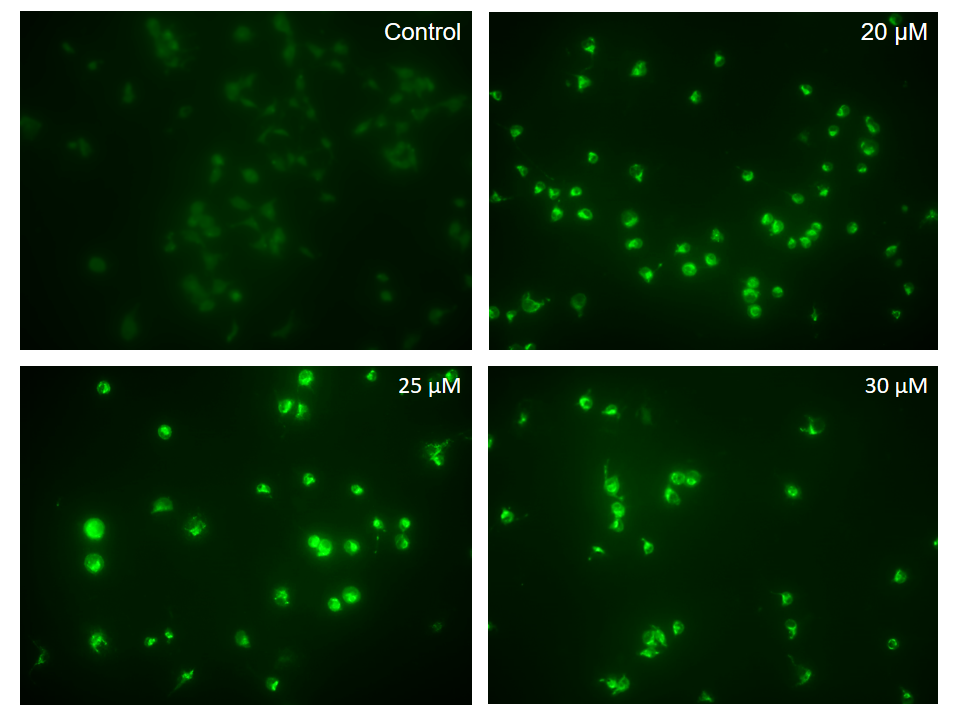
**

**Figure S7.** DHR123 staining was performed to measure mitochondrial ROS generation. HeLa cells were treated with various concentration of PEITC (20, 25 and 30 μM) for 24 hours. Fluorescence microscopy images confirmed the mitochondrial ROS generation in PEITC-treated cells. The image is the representation of three independent experiments.

**
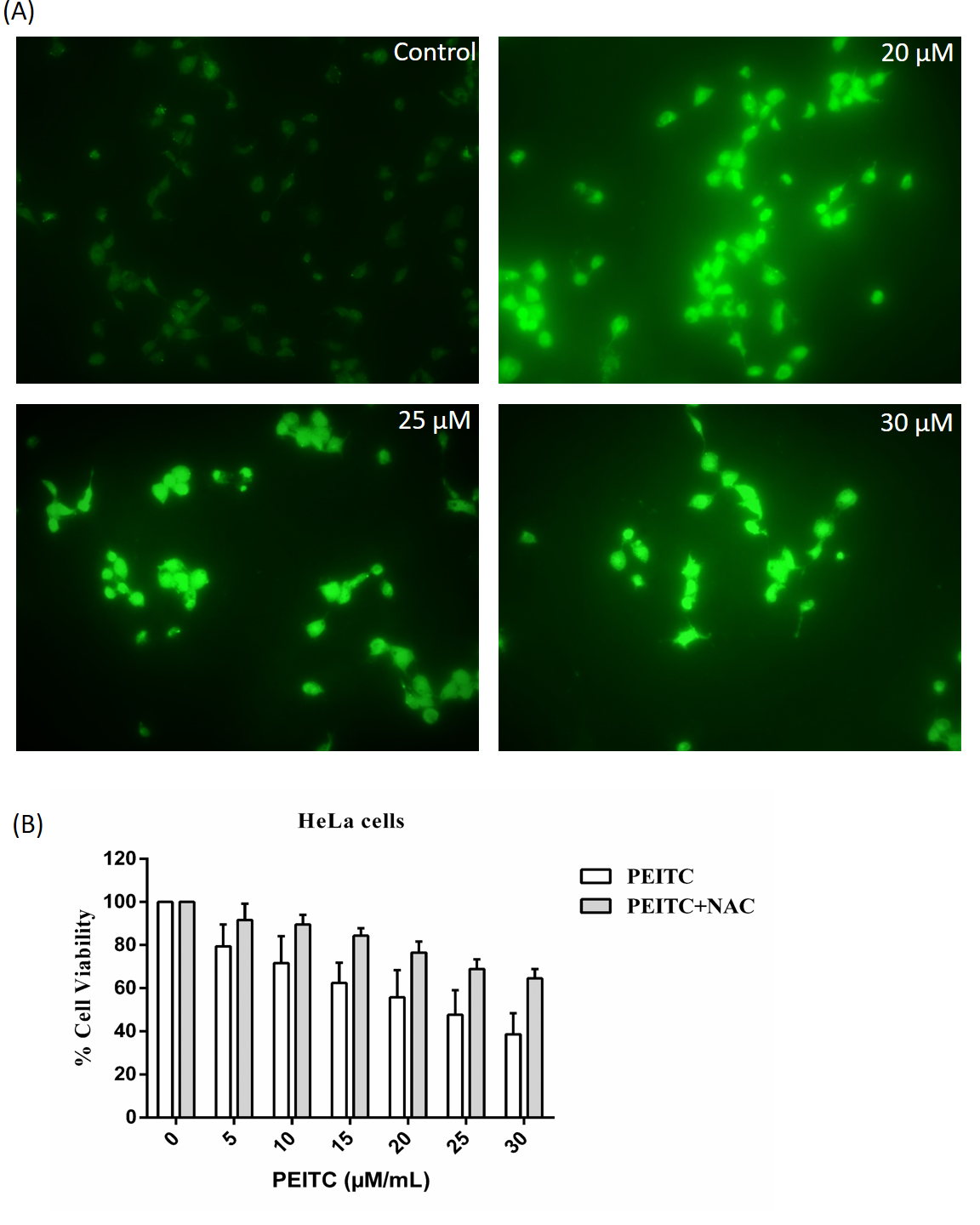
**

**Figure S8.** DCF-DA staining was performed to measure the ROS generation. (A) Fluorescence microscopy images showed significant ROS generation in PEITC-treated HeLa cells. (B) Percent cell viability of PEITC- treated HeLa cells was assessed in presence of NAC (ROS inhibitor) by MTT assay. The data represents mean ±SD of three independent experiments. The image is the representation of three independent experiments.
